# CircKIF4A promotes glioma growth and temozolomide resistance by accelerating glycolysis

**DOI:** 10.1038/s41419-022-05175-z

**Published:** 2022-08-27

**Authors:** Kui Luo, Aiqun Liu, Hao Wu, Qiang Liu, Jin Dai, Yu Liu, Zhifei Wang

**Affiliations:** 1 Department of Neurosurgery, The Third Xiangya Hospital, Central South University, 410013 Changsha, Hunan China; 2 Department of Neurology, School of Clinical Medicine, The First Affiliated Hospital of Guangdong Pharmaceutical University, No.19 Nonglinxia Road, Guangzhou, Guangdong People’s Republic of China

**Keywords:** CNS cancer, Non-coding RNAs

## Abstract

Circular RNAs (circRNAs) are a kind of noncoding RNAs that have different biological functions. CircRNAs play very important parts in the progression of cancers. Nevertheless, the exact mechanism and function of many circRNAs in glioma are not clear. In our study, circKIF4A was identified as a remarkably upregulated circRNA expressed in glioma tissues and cell lines. We performed loss-off function and gain-of-function experiments to inquire into the biological function of circKIF4A in the progression of glioma. We discovered that knockdown of circKIF4A remarkably decreased the proliferation and invasion ability of glioma cells. Moreover, subcutaneous tumorigenesis model and intracranial injection of orthotopic glioma model were established to investigate the functions of circKIF4A in vivo. Suppression of circKIF4A remarkably enhanced the sensitivity of glioma to temozolomide treatment. The glycolysis rate was accelerated by circKIF4A overexpression, which promoted glioma growth and temozolomide resistance. The glycolysis regulating enzyme ALDOA was regulated by circKIF4A through the mechanism of interactivity with miR-335-5p in glioma cells. In a word, our data showed that the upregulation of circKIF4A facilitates glioma progression by means of binding miR-335-5p and upregulating ALDOA expression.

## Introduction

Gliomas are the pervasive primary intracranial tumors, accounting for over 80% of brain malignant tumors [1]. As primary central nervous system tumors that commonly occur in the brain, gliomas are regarded as one of the most invasive diseases to cure due to rapid disease progression and the limitations of surgical treatment [2]. To prolong the survival time of patients with glioma, temozolomide is widely used as an adjuvant chemotherapy agent for patients with glioblastoma, which is very efficient in treating glioma [3–5]. However, nearly half of treated patients with glioma have temozolomide resistance, and all patients ultimately fail treatment [6, 7]. As a result, it remains imperative to shed light on the mechanism of disease progression and temozolomide resistance in glioma [8].

Circular RNAs (circRNAs) are a kind of noncoding RNAs that are shaped by the back-splicing of linear pre-mRNA [9, 10]. CircRNAs can sponge microRNAs (miRNAs) or other molecules (proteins, DNA, etc.) through different molecular mechanisms and play significant parts in controlling the expression of several key genes in cancers [11]. CircRNAs are very steady because of their circular structure and are highly expressed in most mammalian tissues [12]. This characteristic enables the resistance of circRNAs after treatment with RNA exonucleases, such as RNase R [12]. In recent years, more and more circRNAs have been found by high-throughput sequencing and bioinformatic analysis [13]. For instance, cirs-7 is one of the most discussed circRNAs, which spongs miR-7 and promotes cancer growth, invasion, and immune escape in a diversity of malignant tumors [14–19]. The back-spliced circular form of MEG3 noncoding RNA is downregulated in hepatocellular carcinoma and inhibits liver cancer stem cells by reducing telomerase activity [20]. The circular transcript of the RAD18 gene facilitates cancer progression in multiple cancers [21, 22]. IGF1R is upregulated by circGNB1, which has been proven to interact with miR-141-5p to promote tumor growth in breast cancer [23]. CircFBXW7 was found to promote glioma tumorigenesis in glioma by encoding a new 21-kDa protein, which we named it FBXW7-185aa [24]. According to a recent interesting study, circSMARCA5 binds SRSF1 via the GAUGAA RNA Motif to promote cell migration and angiogenesis in multiforme cell glioblastoma [25]. Another circRNA, circSHPRH, inhibits glioma cell proliferation via translating the SHPRH-146aa, which prevents SHPRH protein from being degraded [26]. Several studies have reported that circKIF4A was highly expressed in cancers, which promotes tumors growth, invasion, and metastasis in a diversity of malignancies, including triple-negative breast cancer [27], bladder cancer [28], papillary thyroid cancer [29], and gastric cancer [30]. Nevertheless, the exact mechanism and function of circKIF4A in glioma are not clear.

In this study, circKIF4A was identified as a remarkably upregulated circRNA expressed in glioma tissues and cell lines. We found that the upregulation of circKIF4A increased the proliferation and invasion ability of glioma cells while knockdown of circKIF4A significantly decreased the proliferation and invasion ability. Suppression of circKIF4A remarkably enhanced the sensitivity of glioma to temozolomide treatment. The molecular mechanism of circKIF4A in glioma was uncovered by conducting dual luciferase reporter experiments and RNA immunoprecipitation experiments. The glycolysis rate was accelerated by circKIF4A overexpression, which promoted glioma growth and temozolomide resistance. The glycolysis regulating enzyme ALDOA was regulated by circKIF4A through the mechanism of interactivity with miR-335-5p in glioma cells. The upregulation of circKIF4A facilitates glioma progression by means of binding miR-335-5p and upregulating ALDOA expression. Overall, this study identified the biological function of the circKIF4A-miR-335-5p-ALDOA axis in glioma progression. This result is significant for developing new therapeutic strategies.

## Materials and methods

### Collection of samples and data

Primary glioma tissues and adjacent normal brain tissues were obtained and collected from the patients hospitalized in The Third Xiangya Hospital of Central South University. The clinicopathological characteristics of ten patients with glioma analyzed in this study is shown in Supplemental Table S1. None of the patients experienced either chemotherapy or radiotherapy prior to surgery. All patients with glioma signed the written informed consent before treatment. This study was permitted by the Ethics Committee of the Third Xiangya Hospital and conducted according to the Declaration of Helsinki.

### Cell line source and culture

Cell lines (A172, SHG-44, U251, T98G, and BT325) of glioma cells and the normal cell line HEB were obtained from ATCC. All cell lines were cultured in accordance with the instructions. All cell lines were confirmed by DNA fingerprinting before all experiments.

### RT–qPCR analysis

All qRT–PCR analyses were performed by a SYBR Green qPCR Kit (Takara, Japan). The primers of circKIF4A were F: 5′-GAGGTACCCTGCCTGGATCT-3′ and R: 5′-TGGAATCTCTGTAGGGCACA-3′. The primers of KIF4A were F: 5′-AGCTTCTTTAATCCCGTCTGTG-3′ and R: 5′- GGCCAGAGCCCGTTTCTTT-3′. The primers of GAPDH were F: 5′-GGAGCGAGATCCCTCCAAAAT-3′ and R: 5′-GGCTGTTGTCATACTTCTCATGG-3′. The primers of 18S were F: 5′- TTAATTCCGATAACGAACGAGA-3′and R: 5′-CGCTGAGCCAGTCAGTGTAG-3′.

### RNase R digestion assay

Briefly, 3 µg of total RNA extracted from SHG-44 or A172 glioma cell lines was handled by RNase R (2 U/μg) or dd-water at 37 °C for half an hour. QRT–PCR analysis was used to quantify the remaining RNA solution.

### Actinomycin D digestion assay

At 0-h, 8-h, and 16-h time points, SHG-44 or A172 glioma cell lines were digested by 5 µg/ml actinomycin D (Sigma). Afterward, circular RNA circKIF4A and the linear host gene KIF4A mRNA were examined through qRT–PCR analysis.

### Western blot analysis

Briefly, the protein from cells was extracted with RIPA lysis buffer. The protein concentrations were quantified with a BCA assay kit (Thermo Fisher, USA). Then, all proteins were delivered to a PVDF membrane for two hours. The membrane was incubated overnight with the primary antibody at 4 °C. Then the membrane was exposed to the secondary antibody at room temperature for 1 h. The primary antibodies 1:1000 anti-ALDOA (CST, USA) and 1:3000 anti-beta-actin (Abcam, USA) were applied in our study.

### Cell counting kit-8 (CCK-8) assay

SHG-44 or A172 or U251 glioma cells were accumulated and resuspended. Then, sh-NC (3000 cells/well) and sh-circKIF4A groups (3000 cells/well) were seeded in each well. CCK-8 solution (10 μl) was added to every well and maintained for two hours. The absorbance of the well in each group was measured (450 nm).

### Colony-formation assay

In total, 3 × 10^3^ cells were resuspended and seeded in every well of a 6-well plate. After incubating for 7 days at 37 °C, the colonies were fixed with methanol and then stained with crystal violet (2.5%).

### Transwell assay

All cells (5 × 10^4^) were digested and then resuspended. Cells in each group were transferred to the upper cross-pore compartment (without FBS) and lower cross-pore compartment (containing 20% FBS). All cells stained with crystal violet (2.5%) were counted and imaged.

### Measurement of glucose consumption and lactate production

The Amplex Red Glucose/Glucose Oxidase Assay Kit (Invitrogen, USA) was applied to measure glucose consumption and lactate production. The data were normalized to the total cellular protein amounts.

### Luciferase reporter assay

SHG-44 and A172 glioma cell lines were seeded into wells (3 × 10^3^ cells) of a 96-well plate. The forecasted miR-335-5p binding sites of circKIF4A and the 3’-UTR of ALDOA mRNA were manually mutated. Then, miRNA mimics and reporter vectors (circKIF4A-wt/mut or 3’-UTR of ALDOA-wt/mut) were cotransfected into cells for 48 h.

### RNA immunoprecipitation (RIP)

AGO2 antibody (CST, USA) was used in the assay. The relative expression of circKIF4A, miR-335-5p, and ALDOA mRNA were tested after RNA purification. For the MS2-based immunoprecipitation assays, MS2-Rluc, MS2-circKIF4A, and MS2-circKIF4A-mt plasmid were constructed using pcDNA3.1 vector. Then, 5ug MS2-Rluc, MS2-circKIF4A, or MS2-circKIF4A-mt in each group was transfected to the cells using lipo3000 transfection reagent 72 h before immunoprecipitation assays. The abundance of miR-335-5p was determined after the purification of RNA complexes. Three replicates of each assay were performed in all vitro cell assays.

### Subcutaneous and intracranial xenograft model

BALB/c nude mice (six weeks old) were obtained from SJA Laboratory Animal Company (Hunan, China). Two-hundred microliters of cell suspension (~5 × 10^6^) in PBS was subcutaneously injected into nude mice for constructing the subcutaneous xenograft model. The sizes of tumor were measured every 7 days by electronic calipers for 28 days. The volume of tumor was calculated by means of the formula: Volume = 0.5 × length × width^2^. All mice were sacrificed through cervical dislocation at Day 28 after treatments. Tumor samples were weighed for all groups. Cells (~1 × 10^6^) in 20 μl of serum‐free DMEM were intracranially implanted as previously reported to construct the intracranial xenograft model [31]. Overall survival was assessed by Kaplan–Meier analysis. Excised tumor tissues were fixed, paraffin-embedded, and sectioned. The obtained sections were used to hematoxylin and eosin (HE) staining and immunohistochemistry analysis. The analysis was performed by an assessor who was blind to treatment allocation. The animal experiments were permitted by the Guidelines for the Care and Use of Laboratory Animals and the Medical Ethics Committee of the Third Xiangya Hospital (Changsha, China).

### Statistical analysis

SPSS 24.0 statistical software (USA) was applied to all statistical analyses. The mean ± standard deviation (SD) was applied to report the data. Student’s *t*-test was applied to compare groups. The expression in two matched groups was compared by a paired *t*-test. A *P* < 0.05 was considered significantly.

## Results

### circKIF4A is upregulated with circular RNA characteristics in glioma

**Fig. 1:**
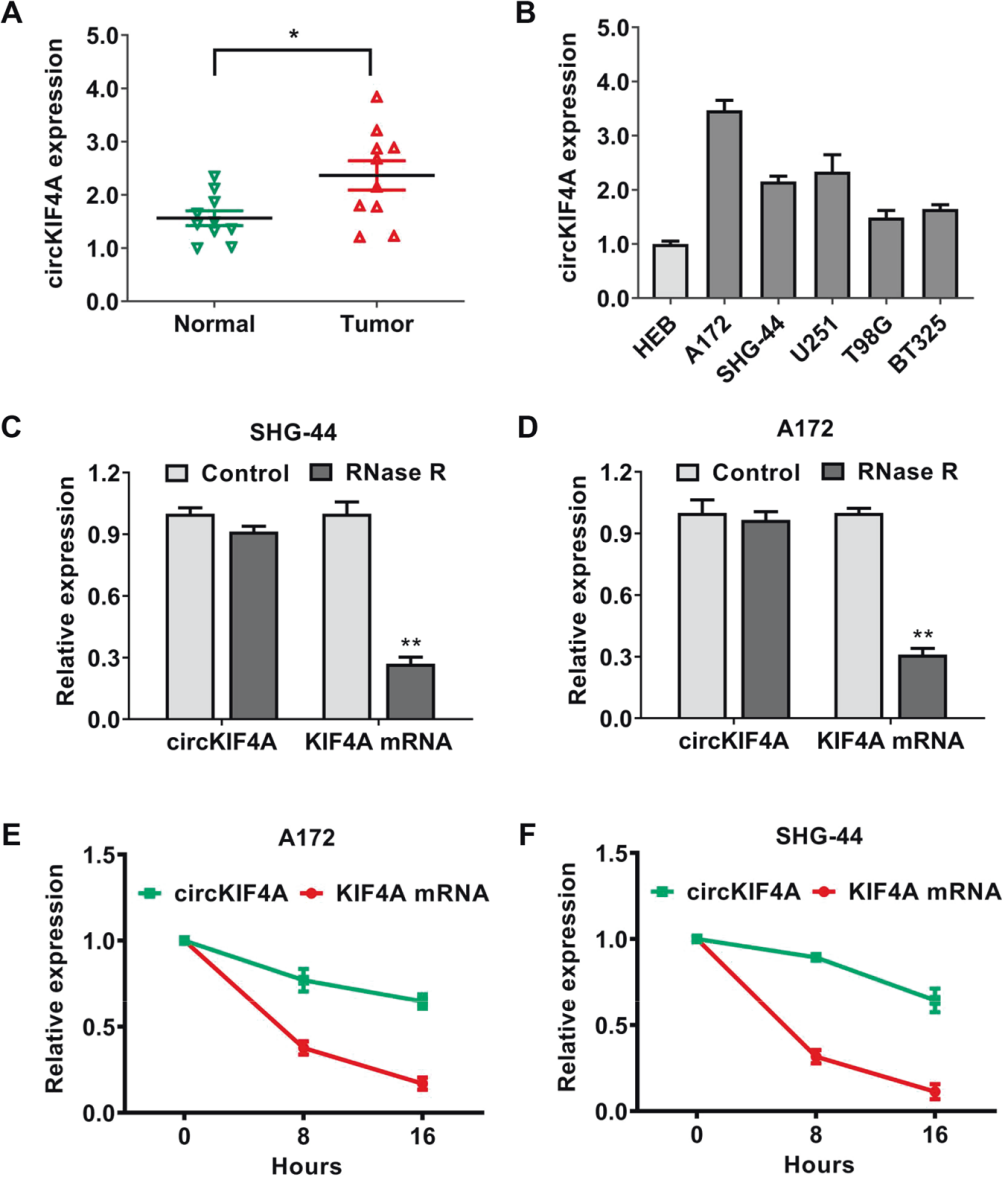
circKIF4A is upregulated with circular RNA characteristics in glioma. **A** The expression of circKIF4A in ten glioma samples and adjacent-matched normal tissues. **B** The expression of circKIF4A in glioma cell lines. **C**, **D** The circular features of circKIF4A were determined by RNase R assays in SHG-44 and A172 glioma cell lines. **E**, **F** As determined by actinomycin D digestive assays, the circular form of KIF4A (circKIF4A) was stable compared to linear mRNA transcripts in SHG-44 and A172 glioma cell lines. *p < 0.05. **p < 0.01. The experiment was repeated three times independently.

Based on the human reference genome (GRCh37/hg19), circKIF4A (hsa_circ_0007255) is located at chrX: 69549254–69553539, which is derived from the 8, 9, and 10 exons of KIF4A (kinesin family member 4A) pre-mRNA. First, we performed qRT–PCR analysis to assess the expression of circKIF4A in glioma tissues and cells. circKIF4A was extremely upregulated in glioma tissues compared to matched normal tissues (Fig. 1A). We validated the expression of circKIF4A in several glioma cell lines. We discovered that the expression of circKIF4A was higher in glioma cell lines than in the normal cell line HEB (Fig. 1B). Next, we determined the circular structure of circKIF4A via RNase R assays and actinomycin D assays. As an RNA with circular features, circKIF4A was stable and resistant to RNase R treatment (Fig. 1C, D). Consistently, circKIF4A had a long half-life time compared to the linear KIF4A mRNA due to the circular form in SHG-44 and A172 glioma cell lines (Fig. 1E, F).

### circKIF4A accelerates glycolysis to promote glioma growth and temozolomide resistance in vitro

**Fig. 2:**
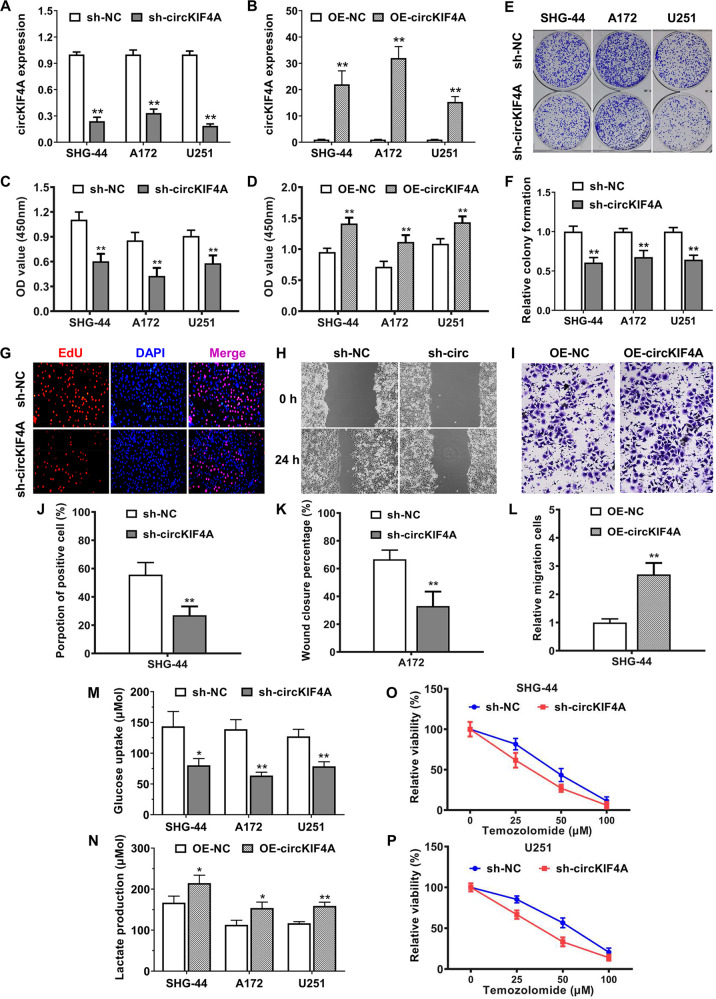
circKIF4A accelerates glycolysis to promote glioma growth and temozolomide resistance in vitro. **A** The effect of shRNAs was validated in SHG-44, A172, and U251 glioma cell lines. **B** The overexpression efficiency of circKIF4A was examined by qPCR analysis. **C**, **D** Knockdown and overexpression of circKIF4A significantly decreased and increased the cell proliferation rate evaluated by CCK-8 assays in SHG-44, A172, and U251 glioma cell lines. **E**, **F** Inhibition of circKIF4A attenuated the colony forming ability of three glioma cell lines assessed by colony-formation experiments. **G**, **J** The cell proliferation rate was evaluated by an EdU assay. **H**, **K** Silencing of circKIF4A decreased the wound closure percentage due to the decreased cell migration ability assessed by a wound-healing assay in the A172 glioma cell line. **I**, **L** Overexpression of circKIF4A increased the relative migration cells because of the increased cell migration ability evaluated by transwell assays in the SHG-44 glioma cell line. **M** The glucose uptake rate of three glioma cell lines was reduced after transfection with shRNA. **N** The lactate production rate of three glioma cell lines was increased after overexpressing circKIF4A. **O**, **P** Inhibition of circKIF4A remarkably enhanced the sensitivity of glioma to temozolomide treatment in SHG-44 and U251 glioma cell lines. *p < 0.05. **p < 0.01. The experiment was repeated three times independently.

Loss-of-function and gain-of-function assays were both conducted for investigating the roles of circKIF4A in the progression of glioma. We designed short-hairpin RNA that aimed at the back-splice junction site of circKIF4A and verified the knockdown efficiency (Fig. 2A). To conduct gain-of-function assays, we established stably overexpressing circKIF4A cell lines via lentiviral infection (Fig. 2B). Knockdown and overexpression of circKIF4A significantly decreased and increased the proliferation rate of three glioma cell lines, respectively (Fig. 2C, D). Inhibition of circKIF4A attenuated the colony forming ability of three glioma cell lines assessed by colony-formation experiments (Fig. 2E, F). Validated by EdU assays, the proliferation rate of glioma cells was decreased after suppression of circKIF4A (Fig. 2G–J). We inquired into the function of circKIF4A in promoting glioma cell metastasis. Silencing of circKIF4A decreased the wound closure percentage in the A172 glioma cell line (Fig. 2H–K). To evaluate the migration rate of SHG-44 glioma cells, circKIF4A was exogenously overexpressed. Overexpression of circKIF4A increased the relative migration cells in the SHG-44 glioma cell line. (Fig. 2I–L). Additionally, circKIF4A could also accelerate glycolysis by increasing glucose uptake and lactate production (Fig. 2M, N). Temozolomide resistance is the major reason for treatment failure in patients with glioma. We discovered that inhibition of circKIF4A significantly enhanced the sensitivity of glioma to temozolomide treatment (Fig. 2O, P).

### circKIF4A facilitates glioma growth and temozolomide resistance in vivo

**Fig. 3:**
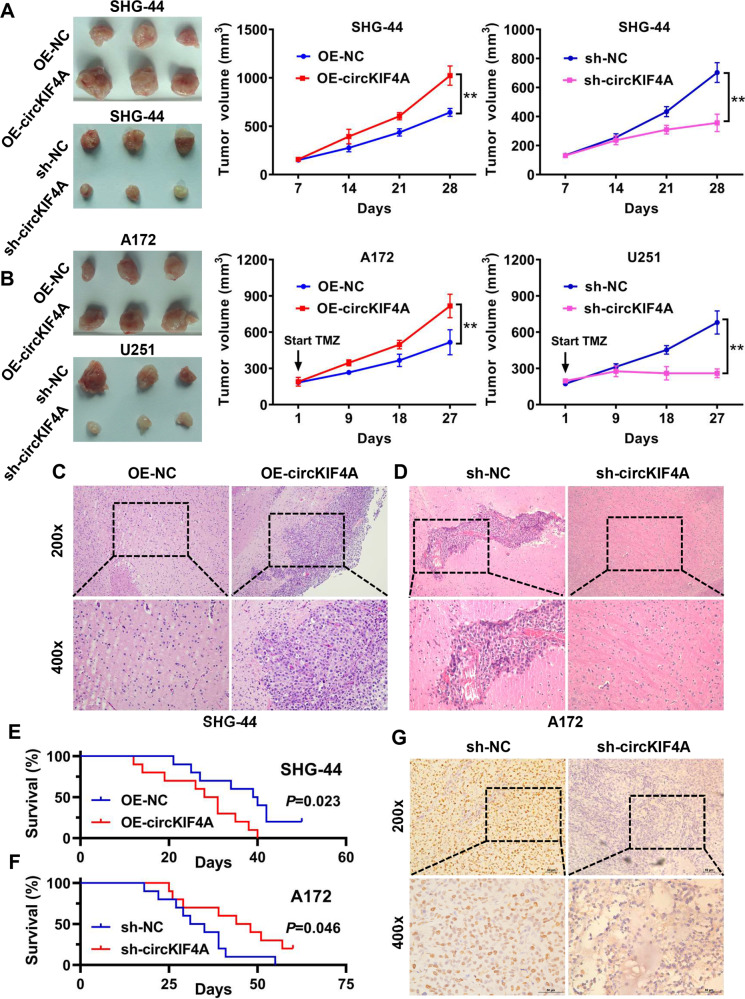
circKIF4A facilitates glioma growth and temozolomide resistance in vivo. **A** Subcutaneous tumorigenesis assays were conducted on nude mice. Overexpression of circKIF4A increased the growth rate and tumor volume. Inhibition of circKIF4A reduced the growth rate and the volume of tumors established by SHG-44 glioma cells. **B** Temozolomide resistance of glioma was evaluated after overexpression and knockdown of circKIF4A in vivo. **C** An orthotopic glioma model was established via intracranial injection of OE-NC or OE-circKIF4A group SHG-44 tumor cells. **D** An orthotopic glioma model was established via intracranial injection of sh-NC or sh-circKIF4A group A172 tumor cells. **E** The overall survival of mice evaluated by Kaplan–Meier survival analysis was decreased after upregulation of circKIF4A in orthotopic glioma models (OE-circKIF4A group). **F** The overall survival of mice evaluated by Kaplan–Meier survival analysis was prolonged after downregulation of circKIF4A in orthotopic glioma models (sh-circKIF4A group). **G** Representative images of Ki-67 (marker of proliferation) immunohistochemical staining in xenograft sections. **p < 0.01. The experiment was repeated three times independently.

We further studied the functions of circKIF4A in vivo. First, overexpression of circKIF4A increased the growth rate and tumor volume in a subcutaneous tumorigenesis assay in nude mice (Fig. 3A). Consistently, inhibition of circKIF4A reduced the growth rate and the volume of tumors established by SHG-44 glioma cells (Fig. 3A). Moreover, we examined the role of circKIF4A in facilitating the temozolomide resistance of glioma in vivo. The tumors established by A172 glioma cells grew well after overexpressing circKIF4A under temozolomide treatment (Fig. 3B). Suppression of circKIF4A significantly increased the sensitivity of glioma to temozolomide treatment in xenografts established by U251 cells (Fig. 3B). Additionally, we established an orthotopic glioma model via intracranial injection of SHG-44 and A172 tumor cells. Our results showed that glioma cell infiltration was lower in the circKIF4A knockdown group and higher in the circKIF4A overexpression group (Fig. 3C, D). The overall survival of mice was also decreased and prolonged after upregulation and downregulation of circKIF4A in orthotopic glioma models (Fig. 3E, F). The proliferation marker Ki67 was used to assess the growth of each tumor. We found that the Ki67 positivity rate was lower in the circKIF4A suppression group (Fig. 3G).

### circKIF4A binds miR-335-5p to promote glioma progression

**Fig. 4:**
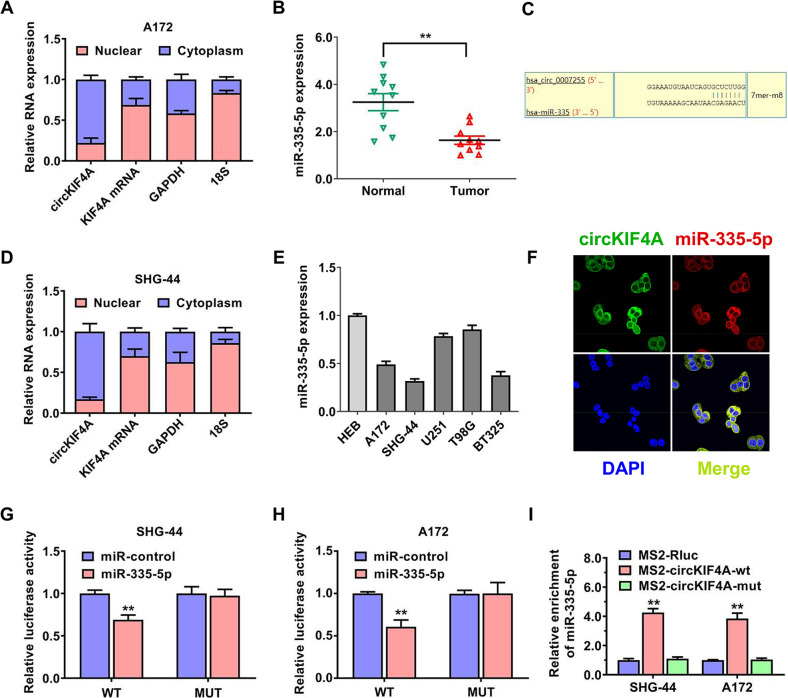
circKIF4A binds miR-335-5p to promote glioma progression. **A** circKIF4A, KIF4A linear mRNA, 18S, and GAPDH in nuclear and cytoplasmic fractions in A172 cells were assessed by RT–qPCR. **B** MiR-355-5p was expressed at low level in the glioma tissues compared to paired normal tissues. **C** Predicted interacting sites of miR-335-5p within the circKIF4A whole sequence. **D** circKIF4A, KIF4A linear mRNA, GAPDH, and 18S in nuclear and cytoplasmic fractions in SHG-44 cells were assessed by RT–qPCR. **E** MiR-355-5p was expressed at lower level in glioma cell lines than in the normal cell line HEB. **F** Confocal laser imaging revealing that circKIF4A and miR-355-5p were colocalized in SHG-44 glioma cells. **G**, **H** Dual luciferase reporter assay of SHG-44 and A172 glioma cell lines transfected with miR-335-5p mimics and circKIF4A wild-type (wt)/mutant (mut) luciferase vectors. **I** MS2-based RNA immunoprecipitation assays were applied to prove the binding of miR-355-5p to circKIF4A in both SHG-44 and A172 glioma cell lines. The relative enrichment of RNA in each group was normalized to the input group. **p < 0.01. The experiment was repeated three times independently.

To inquire into the molecular mechanism of circKIF4A in promoting glioma growth and invasion, we conducted nuclear and cytoplasmic RNA separation experiments. qRT–PCR analysis indicated that circKIF4A was overwhelmingly present in the cytoplasm of cells (Fig. 4A, D). miR-355-5p was predicted to potentially interact with circKIF4A by the circinteractome algorithm with the highest binding score. Based on the binding mode, there is only one predicted binding site of miR-335-5p within the circKIF4A sequence. (Fig. 4C). We collected ten glioma samples and their paired normal tissues, and miR-355-5p was remarkably downregulated in the glioma tissues compared to normal tissues (Fig. 4B). We discovered the expression level of miR-355-5p was also lower in glioma cell lines than in the normal cell line HEB (Fig. 4E). As observed by confocal laser imaging, circKIF4A and miR-355-5p colocalized in SHG-44 glioma cells (Fig. 4F). To evaluate the interactivity of circKIF4A and miR-355-5p, we conducted dual luciferase activity reporter experiments. The relative amount of fluorescence intensity was decreased after transfecting with miR-355-5p in both SHG-44 and A172 glioma cell lines (Fig. 4G, H). Moreover, MS2-based RNA immunoprecipitation assays were applied to prove the binding of miR-355-5p to circKIF4A in both SHG-44 and A172 glioma cell lines (Fig. 4I).

### Glycolysis regulating enzyme ALDOA is the target of miR-335-5p

**Fig. 5:**
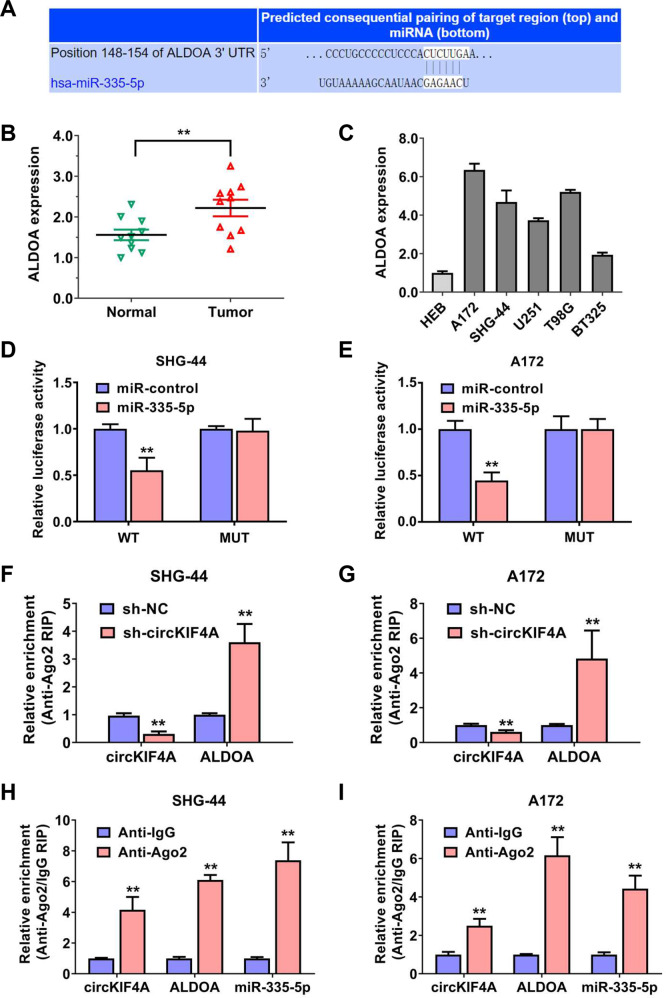
Glycolysis regulating enzyme ALDOA is the target of miR-335-5p. **A** The binding site of miR-335-5p within the 3’-UTR of ALDOA mRNA was predicted by means of the TargetScan database. **B** The expression of ALDOA in glioma and adjacent-matched normal tissues. **C** ALDOA was expressed at higher level in glioma cell lines. **D**, **E** Dual luciferase reporter assays of SHG-44 and A172 glioma cell lines transfected with miR-335-5p mimics and ALDOA mRNA 3’-UTR wild-type (wt)/mutant (mut) luciferase vectors. **F**, **G** Enrichment of AGO2 protein to circKIF4A was reduced while ALDOA was increased after silencing of circKIF4A. **H**, **I** CircKIF4A, miR-355-5p, and ALDOA mRNA were all enriched in the RNA-induced silencing complex in both SHG-44 and A172 glioma cell lines, as assessed by AGO2-related RIP assays. **p < 0.01. The experiment was repeated three times independently.

We further explored the downstream targets of miR-355-5p.Then, we discovered that ALDOA was a potential target, according to the TargetScan algorithm (Fig. 5A). Additionally, the predictive score of ALDOA was the highest in the glycolysis gene set. ALDOA encodes a protein with the function of fructose-bisphosphate aldolase, which is the key enzyme to glycolysis in cells. Using qRT–PCR analysis, ALDOA was remarkably upregulated in glioma compared to adjacent-matched normal tissues (Fig. 5B). Additionally, ALDOA was expressed at higher level in glioma cell lines (Fig. 5C). We next examined the binding of miR-355-5p to ALDOA mRNA 3’-UTR sites. Dual luciferase reporter experiments showed that the amount of fluorescence intensity was decreased after transfecting with miR-355-5p in both SHG-44 and A172 glioma cell lines (Fig. 5D, E). Moreover, the assembly of RISC complexes of ALDOA mRNA was decreased after targeting circKIF4A in both SHG-44 and A172 glioma cell lines (Fig. 5F, G). circKIF4A, miR-355-5p and ALDOA mRNA were all enriched in the RNA-induced silencing complex in both SHG-44 and A172 glioma cell lines, as assessed by AGO2-related RIP assays (Fig. 5H, I)

### circKIF4A promotes glioma progression via the miR-335-5p-ALDOA axis

**Fig. 6:**
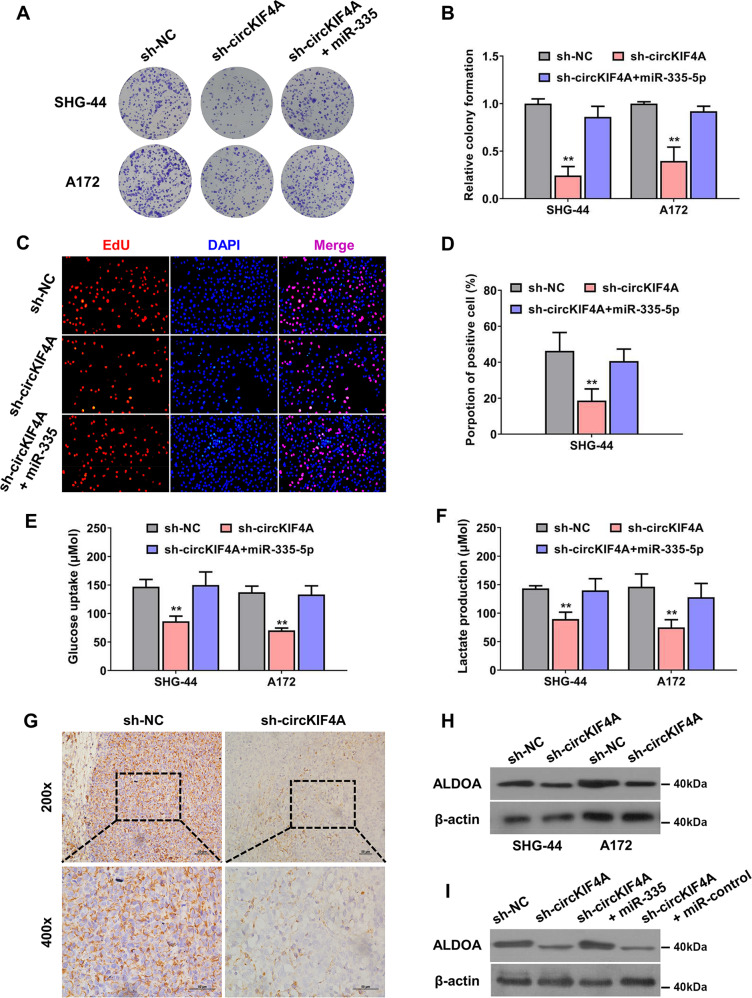
circKIF4A promotes glioma progression via the miR-335-5p-ALDOA axis. **A**, **B** The colony-formation ability of SHG-44 and A172 glioma cells was decreased after knocking down circKIF4A, which was reversed after supplementation with miR-335-5p mimics. **C**, **D** EdU assay was conducted. The proliferation rate was also rescued after transfecting with miR-335-5p mimics when circKIF4A was inhibited in SHG-44 glioma cell lines. **E**, **F** The glucose uptake rate and lactate production speed were reduced after knockdown of circKIF4A, which was reversed by transfection of miR-335-5p mimics. **G** The expression of ALDOA protein was lower in the circKIF4A knockdown group in the mouse xenograft experiments. **H** Western blot assay showing that ALDOA was decreased after the suppression of circKIF4A in SHG-44 and A172 glioma cell lines. **I** Western blot assays revealed that the reduction in ALDOA was rescued by supplementation with miR-335-5p mimics. **p < 0.01. The experiment was repeated three times independently.

We conducted several rescue experiments to validate the function of the circKIF4A-miR-335-5p-ALDOA axis in regulating glioma progression. The colony forming ability of SHG-44 and A172 glioma cells was decreased after knocking down circKIF4A, which was reversed after supplementation with miR-335-5p mimics (Fig. 6A, B). The proliferation rate was rescued after transfecting with miR-335-5p mimics when circKIF4A was inhibited in SHG-44 glioma cell lines (Fig. 6C, D). The glycolysis rate of SHG-44 and A172 glioma cells was decreased after silencing circKIF4A, which was also reversed after supplementation with miR-335-5p mimics (Fig. 6E, F). Then, in the mouse xenograft experiments, the expression of ALDOA protein was lower in the circKIF4A knockdown group (Fig. 6G). According to our results, ALDOA was decreased after the suppression of circKIF4A in SHG-44 and A172 glioma cell lines (Fig. 6H). This effect could be rescued via the introduction of miR-335-5p mimics, which verified the circKIF4A-miR-335-5p-ALDOA axis in glioma (Fig. 6I).

## Discussion

In recent decades, the development and innovation of high-throughput circRNA microarray sequencing and high-computation bioinformatic technology have enabled scientists to identify a lot of circRNAs in different human tissues [32, 33]. Researchers have come to realize that this new kind of noncoding RNA is not junk resulting from false splice products, rather, circRNAs have important biological functions in different cellular processes [9, 34]. CircRNAs can modulate gene expression via several mechanisms, such as binding proteins, interacting with miRNAs, and encoding small molecule peptides [35, 36]. However, to date, there are few studies about the possible molecular mechanism and function of circRNAs in the progression and temozolomide resistance of glioma.

In our study, circKIF4A was identified as a remarkably upregulated circRNA expressed in glioma tissues and cell lines. To reveal the function of circKIF4A in the progression of glioma, we performed loss-off function and gain-of-function experiments in vivo and in vitro. Silencing of circKIF4A remarkably decreased the proliferation and invasion ability of glioma cells. Moreover, we found that suppression of circKIF4A remarkably enhanced the sensitivity of glioma to temozolomide treatment. CircKIF4A overexpression accelerated the glycolysis rate, which promoted glioma growth and temozolomide resistance. In addition, dual luciferase reporter experiments and RNA immunoprecipitation experiments were performed to uncover the mechanism of circKIF4A in glioma. The glycolysis regulating enzyme ALDOA was regulated by circKIF4A through the mechanism of interacting with miR-335-5p in glioma cells.

In accordance with theory of endogenous RNA competition, mRNAs, pseudogenes, lncRNAs and circRNAs can modulate mutually via competitive sponging of shared miRNAs [37]. miR-335-5p has been proven to be a tumor suppressor in lots of malignant tumors. For example, miR-335-5p influences many targets in the BRCA1 cascade, affecting cell apoptosis and proliferation in breast cancer [38]. During the progression of breast cancer, dysregulation of miRNAs may lead to increased tumorigenic potential through negligence of key tumor suppressor signals [39, 40]. Exosomal miR-335 was a tumor suppressor, which could be a new therapeutic strategy in hepatocellular carcinoma [41]. As the downstream target of miR-335-5p, ALDOA promotes human cancer progression by accelerating the glycolysis rate [42, 43]. ALDOA interacts with γ-actin to promote lung cancer metastasis, which could be targeted by an antiretroviral agent that targets HIV integrase (raltegravir) [44]. The poor prognosis of pancreatic cancer predicted by ALDOA is partly because of its regulation of E-cadherin expression. The relevance between ALDOA and E-cadherin was discovered in pancreatic cancer [45]. Our results revealed that circKIF4A and ALDOA acted as endogenous competitive RNAs in glioma.

In a word, our data showed that the upregulation of circKIF4A facilitates glioma progression by means of binding miR-335-5p and upregulating ALDOA expression. Our results are important for developing new therapeutic strategies.

## Supplementary information

Supplemental Table S1

reproducibility checklist aj-checklist

actin1

actin2

ALDOA1

ALDOA2

## Data Availability

The raw data of this paper will be available from the authors.

## References

[1] Ostrom QT, Bauchet L, Davis FG, Deltour I, Fisher JL, Langer CE (2014). The epidemiology of glioma in adults: a “state of the science” review. Neuro Oncol.

[2] Hervey-Jumper SL, Berger MS (2016). Maximizing safe resection of low- and high-grade glioma. J Neurooncol.

[3] Karachi A, Dastmalchi F, Mitchell DA, Rahman M (2018). Temozolomide for immunomodulation in the treatment of glioblastoma. Neuro Oncol.

[4] Bagherian A, Mardani R, Roudi B, Taghizadeh M, Banfshe HR, Ghaderi A (2020). Combination therapy with nanomicellar-curcumin and temozolomide for in vitro therapy of glioblastoma multiforme via Wnt signaling pathways. J Mol Neurosci.

[5] Bagherian A, Roudi B, Masoudian N, Mirzaei H (2021). Anti-glioblastoma effects of nanomicelle-curcumin plus erlotinib. Food Funct.

[6] Lim M, Xia Y, Bettegowda C, Weller M (2018). Current state of immunotherapy for glioblastoma. Nat Rev Clin Oncol.

[7] Meng X, Zhao Y, Han B, Zha C, Zhang Y, Li Z (2020). Dual functionalized brain-targeting nanoinhibitors restrain temozolomide-resistant glioma via attenuating EGFR and MET signaling pathways. Nat Commun.

[8] Khani P, Nasri F, Khani Chamani F, Saeidi F, Sadri Nahand J, Tabibkhooei A (2019). Genetic and epigenetic contribution to astrocytic gliomas pathogenesis. J Neurochem.

[9] Ebbesen KK, Hansen TB, Kjems J (2017). Insights into circular RNA biology. RNA Biol.

[10] Mousavi SM, Derakhshan M, Baharloii F, Dashti F, Mirazimi SMA, Mahjoubin-Tehran M (2022). Non-coding RNAs and glioblastoma: Insight into their roles in metastasis. Mol Ther Oncolytics.

[11] Li J, Sun D, Pu W, Wang J, Peng Y (2020). Circular RNAs in cancer: biogenesis, function, and clinical significance. Trends Cancer.

[12] Kristensen LS, Andersen MS, Stagsted LVW, Ebbesen KK, Hansen TB, Kjems J (2019). The biogenesis, biology and characterization of circular RNAs. Nat Rev Genet.

[13] Gao Y, Shang S, Guo S, Li X, Zhou H, Liu H (2021). Lnc2Cancer 3.0: an updated resource for experimentally supported lncRNA/circRNA cancer associations and web tools based on RNA-seq and scRNA-seq data. Nucleic Acids Res.

[14] Memczak S, Jens M, Elefsinioti A, Torti F, Krueger J, Rybak A (2013). Circular RNAs are a large class of animal RNAs with regulatory potency. Nature.

[15] Hansen TB, Kjems J, Damgaard CK (2013). Circular RNA and miR-7 in cancer. Cancer Res.

[16] Rahmati Y, Asemani Y, Aghamiri S, Ezzatifar F, Najafi S (2021). CiRS-7/CDR1as; An oncogenic circular RNA as a potential cancer biomarker. Pathol Res Pr.

[17] Zou Y, Zheng S, Deng X, Yang A, Xie X, Tang H (2019). The role of circular RNA CDR1as/ciRS-7 in regulating tumor microenvironment: a pan-cancer analysis. Biomolecules.

[18] Kristensen LS, Ebbesen KK, Sokol M, Jakobsen T, Korsgaard U, Eriksen AC (2020). Spatial expression analyses of the putative oncogene ciRS-7 in cancer reshape the microRNA sponge theory. Nat Commun.

[19] Zou Y, Zheng S, Deng X, Yang A, Kong Y, Kohansal M (2020). Diagnostic and prognostic value of circular RNA CDR1as/ciRS-7 for solid tumours: A systematic review and meta-analysis. J Cell Mol Med.

[20] Jiang X, Xing L, Chen Y, Qin R, Song S, Lu Y (2021). CircMEG3 inhibits telomerase activity by reducing Cbf5 in human liver cancer stem cells. Mol Ther Nucleic Acids.

[21] Zou Y, Zheng S, Xiao W, Xie X, Yang A, Gao G (2019). circRAD18 sponges miR-208a/3164 to promote triple-negative breast cancer progression through regulating IGF1 and FGF2 expression. Carcinogenesis.

[22] Zang H, Li Y, Zhang X, Huang G (2020). Knockdown of circRAD18 mitigates breast cancer progression through the regulation of miR-613/HK2 axis. Cancer Manag Res.

[23] Liu P, Zou Y, Li X, Yang A, Ye F, Zhang J (2020). circGNB1 facilitates triple-negative breast cancer progression by regulating miR-141-5p-IGF1R axis. Front Genet.

[24] Yang Y, Gao X, Zhang M, Yan S, Sun C, Xiao F (2018). Novel role of FBXW7 circular RNA in repressing glioma tumorigenesis. J Natl Cancer Inst.

[25] Barbagallo D, Caponnetto A, Barbagallo C, Battaglia R, Mirabella F, Brex D (2021). The GAUGAA motif is responsible for the binding between circSMARCA5 and SRSF1 and related downstream effects on glioblastoma multiforme cell migration and angiogenic potential. Int J Mol Sci.

[26] Zhang M, Huang N, Yang X, Luo J, Yan S, Xiao F (2018). A novel protein encoded by the circular form of the SHPRH gene suppresses glioma tumorigenesis. Oncogene.

[27] Tang H, Huang X, Wang J, Yang L, Kong Y, Gao G (2019). circKIF4A acts as a prognostic factor and mediator to regulate the progression of triple-negative breast cancer. Mol Cancer.

[28] Shi YR, Wu Z, Xiong K, Liao QJ, Ye X, Yang P (2020). Circular RNA circKIF4A sponges miR-375/1231 to promote bladder cancer progression by upregulating NOTCH2 expression. Front Pharm.

[29] Chen W, Fu J, Chen Y, Li Y, Ning L, Huang D (2021). Circular RNA circKIF4A facilitates the malignant progression and suppresses ferroptosis by sponging miR-1231 and upregulating GPX4 in papillary thyroid cancer. Aging (Albany NY).

[30] Yan H, Han L, He N, Li R, He S (2022). Upregulated circular RNA KIF4A promotes cell migration and invasion by regulating microRNA-144-3p/EZH2 axis in gastric cancer. J Oncol.

[31] Feng Z, Zhou W, Wang J, Qi Q, Han M, Kong Y (2020). Reduced expression of proteolipid protein 2 increases ER stress-induced apoptosis and autophagy in glioblastoma. J Cell Mol Med.

[32] Vo JN, Cieslik M, Zhang Y, Shukla S, Xiao L, Zhang Y (2019). The landscape of circular RNA in. Cancer Cell..

[33] Lu D, Thum T (2019). RNA-based diagnostic and therapeutic strategies for cardiovascular disease. Nat Rev Cardiol.

[34] Dashti F, Mirazimi SMA, Rabiei N, Fathazam R, Rabiei N, Piroozmand H (2021). The role of non-coding RNAs in chemotherapy for gastrointestinal cancers. Mol Ther Nucleic Acids.

[35] Chen LL (2020). The expanding regulatory mechanisms and cellular functions of circular RNAs. Nat Rev Mol Cell Biol.

[36] Razavi ZS, Tajiknia V, Majidi S, Ghandali M, Mirzaei HR, Rahimian N (2021). Gynecologic cancers and non-coding RNAs: epigenetic regulators with emerging roles. Crit Rev Oncol Hematol.

[37] Tay Y, Rinn J, Pandolfi PP (2014). The multilayered complexity of ceRNA crosstalk and competition. Nature.

[38] Heyn H, Engelmann M, Schreek S, Ahrens P, Lehmann U, Kreipe H (2011). MicroRNA miR-335 is crucial for the BRCA1 regulatory cascade in breast cancer development. Int J Cancer.

[39] Zhang S, Kim K, Jin UH, Pfent C, Cao H, Amendt B (2012). Aryl hydrocarbon receptor agonists induce microRNA-335 expression and inhibit lung metastasis of estrogen receptor negative breast cancer cells. Mol Cancer Ther.

[40] Xie X, Wang J, Shi D, Zou Y, Xiong Z, Li X (2019). Identification of a 4-mRNA metastasis-related prognostic signature for patients with breast cancer. J Cell Mol Med.

[41] Wang F, Li L, Piontek K, Sakaguchi M, Selaru FM (2018). Exosome miR-335 as a novel therapeutic strategy in hepatocellular carcinoma. Hepatology..

[42] Chang YC, Yang YC, Tien CP, Yang CJ, Hsiao M (2018). Roles of aldolase family genes in human cancers and diseases. Trends Endocrinol Metab.

[43] Mirzaei H, Hamblin MR (2020). Regulation of glycolysis by non-coding RNAs in cancer: switching on the Warburg effect. Mol Ther Oncolytics.

[44] Chang YC, Chiou J, Yang YF, Su CY, Lin YF, Yang CN (2019). Therapeutic targeting of aldolase A interactions inhibits lung cancer metastasis and prolongs survival. Cancer Res.

[45] Ji S, Zhang B, Liu J, Qin Y, Liang C, Shi S (2016). ALDOA functions as an oncogene in the highly metastatic pancreatic cancer. Cancer Lett.

