# Supplementary material for: CircKIF4A promotes glioma growth and temozolomide resistance by accelerating glycolysis
Source: Cell Death Dis. 2022 Aug 27;13(8):740. doi: 10.1038/s41419-022-05175-z (PMC9420136; doi:10.1038/s41419-022-05175-z)
Supplement: Supplementary file 1 — Supplemental Table S1 [file 41419_2022_5175_MOESM1_ESM.docx]

**Supplemental Table S1** Clinicopathological characteristics of ten patients with glioma analyzed in this study. KPS score: Karnofsky Performance Scale status score; GTR: gross total resection; PR: partial resection.

| Clinicopathological characteristics | Cases No. | circKIF4A Expression level | | P value |
| --- | --- | --- | --- | --- |
|  |  | high | low | (* p<0.05) |
| **Age (years)** |  |  |  |  |
| ≥60 | 6 | 3 | 3 | 0.999 |
| <60 | 4 | 2 | 2 |  |
| **Gender** |  |  |  |  |
| Female | 3 | 1 | 2 | 0.490 |
| Male | 7 | 4 | 3 |  |
| **Smoking status** |  |  |  |  |
| No | 8 | 4 | 4 | 0.999 |
| Yes | 2 | 1 | 1 |  |
| **KPS score** |  |  |  |  |
| ≤80 | 6 | 4 | 2 | 0.197 |
| ＞80 | 4 | 1 | 3 |  |
| **WHO grade** |  |  |  |  |
| Ⅰ-Ⅱ | 5 | 1 | 4 | 0.058 |
| Ⅲ-Ⅳ | 5 | 4 | 1 |  |
| **Surgery** |  |  |  |  |
| GTR | 5 | 3 | 2 | 0.527 |
| PR | 5 | 2 | 3 |  |
