# Supplementary figures and images for: CircKIF4A promotes glioma growth and temozolomide resistance by accelerating glycolysis
Source: Cell Death Dis. 2022 Aug 27;13(8):740. doi: 10.1038/s41419-022-05175-z (PMC9420136; doi:10.1038/s41419-022-05175-z)

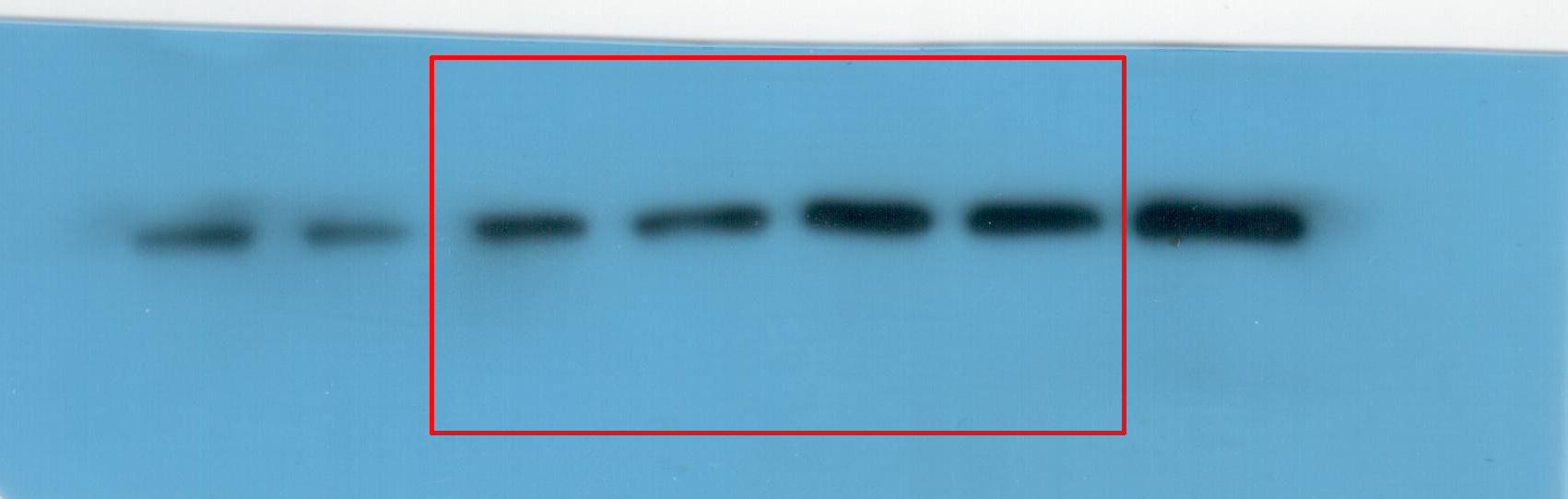

Supplement: Supplementary file 3 — actin1 [file 41419_2022_5175_MOESM3_ESM.tif]

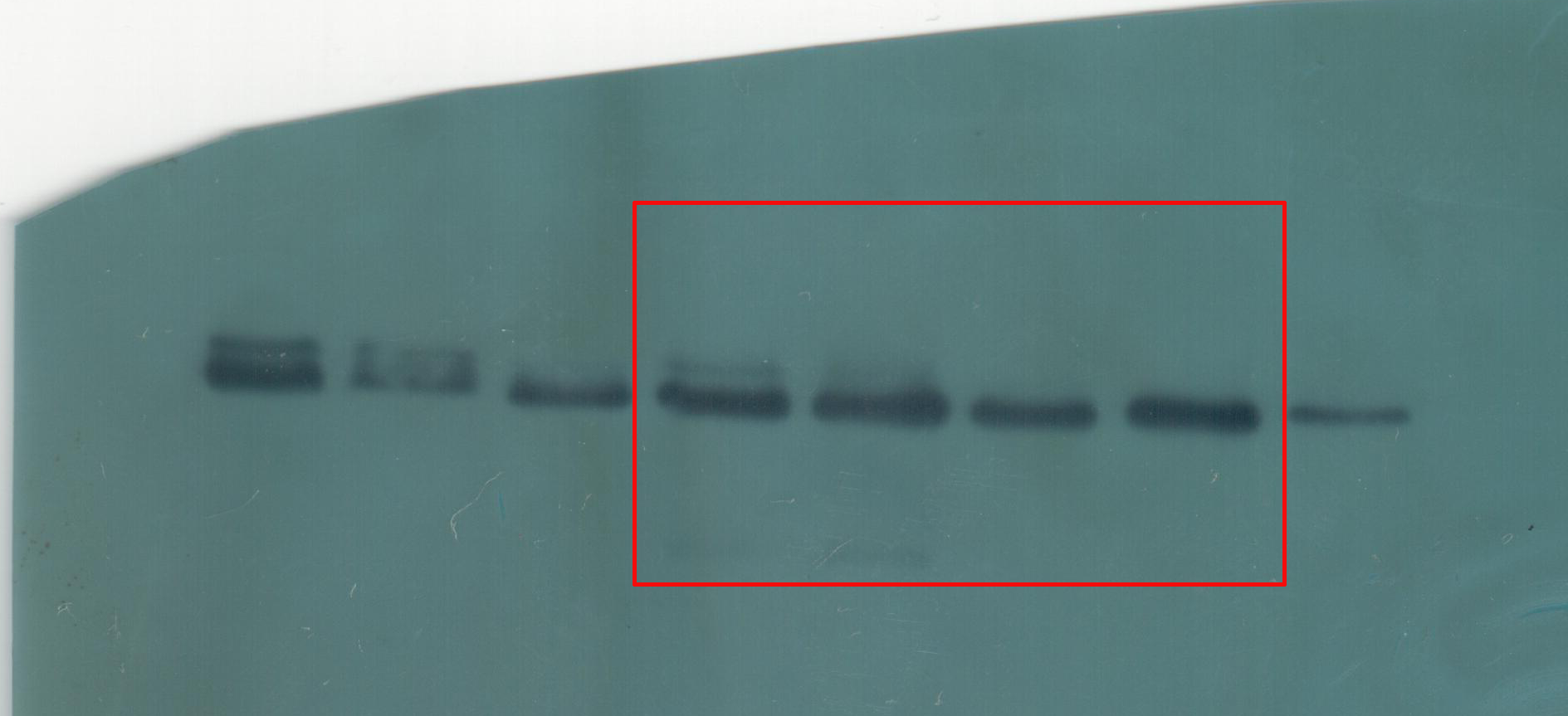

Supplement: Supplementary file 4 — actin2 [file 41419_2022_5175_MOESM4_ESM.tif]

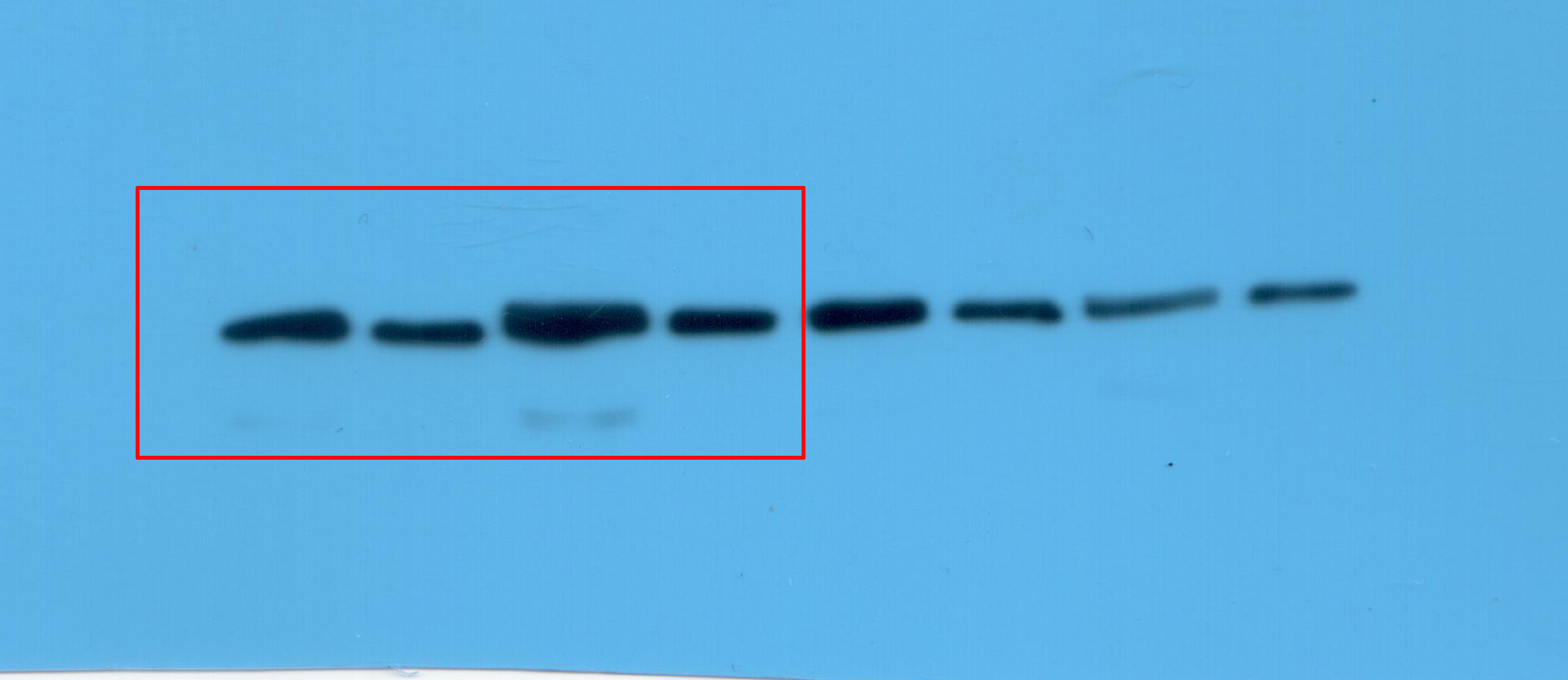

Supplement: Supplementary file 5 — ALDOA1 [file 41419_2022_5175_MOESM5_ESM.tif]

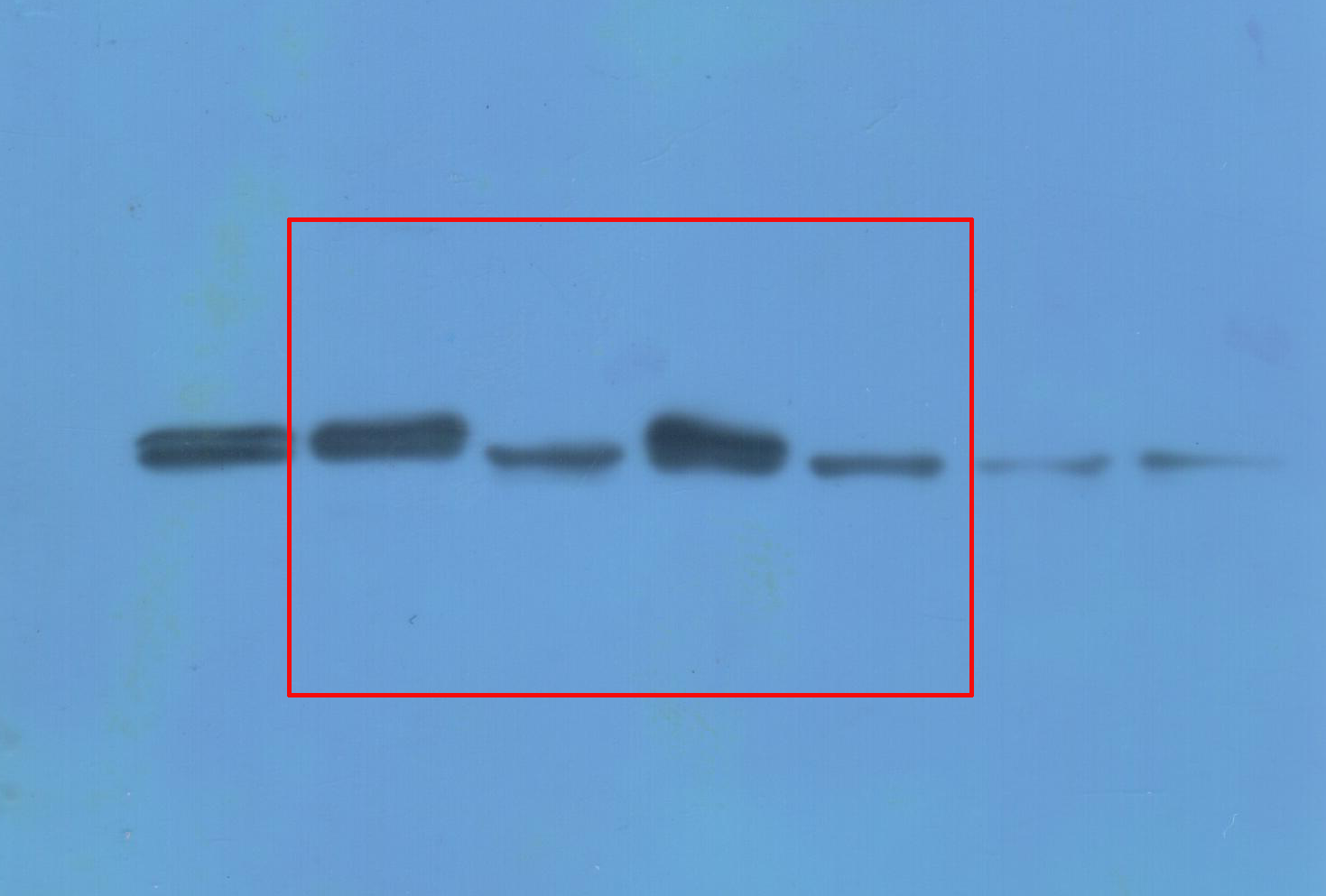

Supplement: Supplementary file 6 — ALDOA2 [file 41419_2022_5175_MOESM6_ESM.tif]
